# Health Resorts

**Published:** 1902-06-21

**Authors:** 


### HEALTH RESORTS.

### EASTBOURNE.

Eastbourne is a town of about 50,000, situated on the south-easterly slope of the south Downs, which at the back of the town rise to a considerable elevation, terminating seawards in the striking cliffs of Beachy Head.

The greater part of Eastbourne, in fact all the upper portion, stands upon chalk, but in the centre of the town there is a certain amount of clay soil and a strip of upper greensand. The lower and eastern end stands chiefly upon alluvium and on the beach. Thus it will be seen that so far as soil conditions are concerned there are great differences between the different parts of the town, and these are accentuated by the fact that the levels in those portions which are covered with houses range from about 150 feet above sea-level in the west to 4 feet below high-water mark in the east. The highest point in the borough, on the Downs, is about 590 feet above the sea. Moreover, the splendid sea-walls and terraced parades, with the fine houses and hotels, by which they are backed, stand considerably higher than a good deal of the land behind, so that, as anyone may see who takes the trouble to look down into the town from the more elevated surroundings, there is much less atmospheric movement in the central "shopping" part of the town than there is, either along the "front" or in the more elevated districts to the north and west, where the climate may be described as distinctly bracing at all times of the year. Eastbourne is an especially sunny spot. Taking the whole year the number of hours of "bright sunshine" is given as 1876·3 against 1338·1 for London. Such a comparison as this, however, does not give the whole truth. The point of importance is as to winter sun. In this respect Eastbourne stands very high on the list, and it is no doubt largely to this that its growing popularity as a winter resort is due.

The special charm of Eastbourne lies in its splendid sea-walls and parades, its beautiful Devonshire Park, the width of its tree-lined streets, the general openness of its plan, and the striking rapidity with which rain dries up so that outdoor exercise can be taken all the year round. Moreover it is a town of sufficient size to give it a social life of its own, independent of its holiday population, and to maintain a good supply of public amusements, while excursions can be made and outside interests discovered in every direction.

Eastbourne also possesses the great advantage of having been built from the beginning upon a well thought-out plan, and of possessing an active and well organised public health department. The birth-rate of the borough as given in a recent report was 20·45; the death-rate, including all deaths, 11·45, but excluding deaths of visitors, 10·49 per 1,000 living; while the death-rate from the seven principal zymotic diseases was 0·77 per 1,000 living.

The town is well provided with sanitary machinery There are (1) a general isolation hospital for the reception of scarlet fever, diphtheria, and enteric fever; (2) a cottage standing in its own grounds used for "shelter" and quarantine purposes; and (3) a separate hospital for small-pox situated half a mile from the nearest house. As showing how energetic the sanitary department is in encouraging the isolation of infectious cases it may be mentioned that of the cases of scarlet fever, enteric fever, and diphtheria which were notified in a recent year 92·1 per cent. were removed to hospital.

The drainage is good. Naturally there has been some trouble with the low-lying portion of the town, but that has been got over by the use of Shone's Ejectors. All the sewage except that from the isolation hospital, which is destroyed by a cremator, passes untreated into the sea a mile to the east of the parade. A careful system of house inspection is maintained, and certificates are given to those houses which attain a given standard of sanitary completeness. In regard to these certificates visitors should take heed to see what date they bear. They are not intended to hold good for more than three years.

Take it altogether, Eastbourne may be regarded as an admirable place for recuperation after illness, for the treatment of many chronic disorders, and as a pleasant sunn residence. The difference between the higher and the lower parts of the town must not be forgotten.

### WOODHALL SPA.

At Woodhall Spa, which is situated on the Horncastle Branch of the Great Northern Railway we have a watering place which is a health resort pure and simple, a place to which people go for a "cure" almost in the German sense of the word. For Woodhall Spa depends for its popularity upon its mineral water, which in regard to certain of its constituents is of peculiar and, so far as England is concerned, of unique composition. This water is classed amongst the cold muriated or common salt waters, but it is peculiar in containing certain minute quantities of bromides and iodides to which many of its special virtues are wont to be attributed. It must be borne in mind, however, that even were one to put on one side the therapeutic efficacy of the bromides and iodides, the quantity of which undoubtedly is but small, Woodhall Spa stands high among the muriated waters whose utility in treatment is well known. Perhaps among the foreign springs this water may be most nearly compared with that of the Elisabethquelle of Kreuznach, and, as at that place, its efficacy is seen in the treatment of strumous and ricketty affections, of many forms of catarrh of the respiratory and digestive tract, and especially of rheumatic, rheumatoid, and gouty affections. Many uterine cases also are found to receive benefit, possibly as a result of the beneficial action of the waters on the catarrhal troubles complications which so often give rise to the leading symptoms in these cases. Woodhall Spa is situated between the Wolds and the Fen, in moorland country, covered with heather, and broom, and gorse, and with a sandy subsoil, through which water quickly percolates. The rainfall is said to be less than in any other part of England. The domestic water supply is provided by the Horncastle Water Company, and comes from the limestone rocks at Cawkwell on the Wolds, whence it is conveyed in iron pipes. The drains are on the separate system, the house drainage and the surface water being separately dealt with. The sewage being dealt with on the Shone system, it has been possible to arrange for a good fall, and this, together with the absence of cesspools, has no doubt had much to do with the freedom of the place from epidemics. Woodhall Spa may undoubtedly be described as a fairly dull place. Still it is by no means devoid of amusement. A band plays several times a day during the season; there are tennis courts, a croquet ground, bowling green, and a golf club; fishing and a little boating can be had; and for those who cycle, or ride or drive, or even to those who merely pedestrianise—decaying art though that be—there are plenty of places of interest within reach. It must, moreover, be borne in mind that at a place like this, those who are undergoing a course rarely find life dull. As at the German spas time is fairly well filled up by the "cure." In regard to the course usually gone through by the patients, we may say that it is customary for those who drink the water to take it in gradually increasing doses; the daily quantity for an adult running from 10 to 30 ounces. The system is to sip the water slowly; those who are able, walking about gently all the time, a plan which might usefully be imitated at other spas, for undoubtedly some of the discomfort which at times follows the imbibition of mineral waters is due to gulping down too large a quantity at a time. It is sometimes found that the water causes constipation at first, but when full doses are arrived at satisfactory evacuations are produced, while, as is generally the case with similar waters, the quantity of urine is increased. For external use the water is applied in the form of full baths, sitz baths, massage douches, nasal sprays, and general or local vapour baths, while other accessory methods such as pine baths and the Dowsing radiant heat and light baths have also been established. The baths are open from the end of March to the end of October.

